# Performance evaluation of two SiPMs arrays coupled to pixelated scintillations for PET/MR applications

**DOI:** 10.1186/2197-7364-2-S1-A8

**Published:** 2015-05-18

**Authors:** Maria Georgiou, Tomasz Szczesniak, Martyna Grodzicka, Eleftherios Fysikopoulos, George Loudos

**Affiliations:** Technological Educational Institute of Athens, Athens, Greece; National Centre for Nuclear Research (NCBJ), Swierk, Poland

The purpose of this study is to investigate the behavior of the new generation of SensL SiPM arrays, ArrayB and ArrayM, for PET/MR applications. The evaluation of the SiPMs performance underwent with pixelated GaGG and BGO scintillators with 2x2x5 mm^3^ pixel size and various coupling schemes for 511keV and 662keV energies. To acquire raw images, we used a symmetric resistive voltage division network and the 4x4 SiPMs anodes reduced to 2X and 2Y position signals. A FPGA Spartan 6 LX150T was used for triggering and digital processing of the pulses acquired using free running ADCs. The first step of our work was optimization of the bias voltage of the two SiPM arrays. The optimal bias voltage is a tradeoff between high photon detection efficiency and low excess noise factor. The SiPMs were coupled to homogeneous 16x16x10 mm^3^ CsITl scintillator and irradiated with ^137^Cs source. The energy resolution calculated in overvoltages between 26.5V and 30.5V for ArrayB and between 28.3V and 31.3V for ArrayM. The bias voltage with the best energy resolution was used for the evaluation of the SiPMs. The clear visualization of the GaGG and BGO crystal elements is expressed quantitatively by the mean peak-valley ratio of a horizontal profile in the raw images. The best optical coupling for the BGO was 2 mm thick glass. In the case of the GaGG 1 mm thick glass was adequate. The mean energy resolution of the GaGG scintillator is about two times better than the BGO for both type of SiPMs, after applying correction for their non-linear response. The low light output of the BGO scintillator in comparison with the GaGG as well as different emission spectra and the different PDE of the two types of SiPMs explain the differences in the behavior of the tested SiPMs arrays.

